# Identification and analysis of oxidative stress‐related genes in hypoxic‐ischemic brain damage using bioinformatics and experimental verification

**DOI:** 10.1002/iid3.70000

**Published:** 2024-08-22

**Authors:** Ni Jin, Sha Sha, Yanghao Ruan, Ying Ouyang

**Affiliations:** ^1^ Sun Yat‐sen Memorial Hospital Sun Yat‐sen University Guangzhou China; ^2^ Fifth Affiliated Hospital of Sun Yat‐sen University Zhuhai China

**Keywords:** bioinformatics analysis, hypoxic‐ischemic brain damage (HIBD), immune infiltration, oxidative stress (OS), oxidative stress‐related differentially expressed genes (OS‐DEGs)

## Abstract

**Background:**

Oxidative stress (OS) plays a major role in the progress of hypoxic‐ischemic brain damage (HIBD). This study aimed to investigate OS‐related genes and their underlying molecular mechanisms in neonatal HIBD.

**Methods:**

Microarray data sets were acquired from the Gene Expression Omnibus (GEO) database to screen the differentially expressed genes (DEGs) between control samples and HIBD samples. OS‐related genes were drawn from GeneCards and OS‐DEGs in HIBD were obtained by intersecting with the DEGs. Subsequently, Gene Ontology (GO) and Kyoto Encyclopedia of Genes and Genomes (KEGG), and Gene Set Enrichment Analysis (GSEA) were conducted to determine the underlying mechanisms and functions of OS‐DEGs in HIBD. Moreover, the hub genes were screened using the protein−protein interaction network and identified in the GSE144456 data set. CIBERSORT was then performed to evaluate the expression of immunocytes in each sample and perform a correlation analysis of the optimal OS‐DEGs and immunocytes. Finally, quantitative reverse transcription polymerase chain reaction (RT‐qPCR) and immunohistochemistry were performed to validate the expression levels of the optimal OS‐DEGs.

**Results:**

In total, 93 OS‐DEGs were identified. GO, KEGG, and GSEA enrichment analyses indicated that these genes were predominantly enriched in OS and inflammation. Four OS‐related biomarker genes (Jun, Fos, Tlr2, and Atf3) were identified and verified. CIBERSORT analysis revealed the dysregulation of six types of immune cells in the HIBD group. Moreover, 47 drugs that might target four OS‐related biomarker genes were screened. Eventually, RT‐qPCR and immunohistochemistry results for rat samples further validated the expression levels of Fos, Tlr2, and Atf3.

**Conclusions:**

Fos, Tlr2 and Atf3 are potential OS‐related biomarkers of HIBD progression. The mechanisms of OS are associated with those of neonatal HIBD.

## Graphical abstract

This research was the first to explore the underlying oxidative stress (OS)‐related genes in hypoxic‐ischemic brain damage (HIBD) and their correlation with immunocytes infiltration. Three potential OS‐related marker genes (Fos, Tlr2, and Atf3) associated with HIBD were identified using bioinformatics analysis and experimentally validated in rat samples.

## 1 INTRODUCTION

Neonatal hypoxic‐ischemic brain damage (HIBD) is a major contributors to neonatal mortality and neurologic impairment.^1^ Moreover, HIBD can result in different levels of lifelong disability, such as behavioral disorders, mental retardation, cerebral palsy, and epilepsy.^2^ Mild hypothermia therapy within 6 h after birth is the most common and basic treatment for neonatal HIBD, keeping the whole body at 33.5°C for 72 h.^3^ Nevertheless, therapeutic hypothermia is only partially effective and does not provide full neuroprotection.^4^ Even after therapeutic hypothermia, there is some infants with moderate or severe perinatal asphyxia experience end up to irreversible neurological sequelae or death.^5^ Thus, exploring the potential biological mechanisms of HIBD development and recognizing the underlying therapeutic targets in neonatal patients are essential.

HIBD pathophysiology can be classified into five main mechanisms, closely correlated one to the others: oxidative stress (OS), inflammation, excitotoxicity, intracellular Ca^2+^ accumulation, and mitochondrial dysfunction.^6^ Owing to the high concentrations of non‐saturated fatty acids, metal‐catalyzed free radicals, and small amounts of antioxidant enzymes in immature brain tissue, the neonatal brain demands higher levels of oxygen, making it particularly sensitive to OS.^7^, ^8^ OS is one of the major mechanisms of HIBD pathophysiology and is caused by impaired mitochondrial function, resulting in the excess accumulation of reactive oxygen species (ROS).^9^ This process is initiated by excitotoxicity caused by the excessive release of excitatory neurotransmitters such as glutamate. Normally, the pro‐oxidants and antioxidants with the organism maintain a balance, and the release of ROS can be immediately eliminated by glutathione (GSH) peroxidase and superoxide dismutase.^10^ Following the hypoxia and ischemia insult, ROS cannot be cleared by antioxidant enzymes with a timely manner, causing a severe damage to proteins, lipids, and nucleic acids, leading to protein and lipid oxidation and DNA degeneration, and eventually leading to neuronal cell death.^11^ Studies have shown that reducing OS can attenuate brain damage and improve neurobehavioral performance in rats with HIBD.^12^, ^13^ However, the potential role and mechanisms of OS in the pathogenesis of neonatal HIBD have not been fully elucidated.

With the popularization of microarray technology and high‐throughput sequencing, bioinformatics methods can be used to reveal the pathogenesis of diseases and new therapeutic targets. This study aimed to identify the differentially expression genes (DEGs) associated with OS in HIBD, and explore the correlation between immune cells and OS‐related genes using bioinformatics technology, providing a novel viewpoint for the mechanisms and molecular targeted therapy of neonatal HIBD.

## 2 MATERIALS AND METHODS

### 2.1 Data sources and pretreatment

Two mRNA expression profiles of GSE23317 and GSE144456^14^ were obtained from the Gene Expression Omnibus (GEO) database (http://www.ncbi.nlm.nih.gov/geo). The GSE23317 data set was used as the training set and the GSE144456 was used as the validation set. The first data set, GSE23317, was derived from the GPL6885 sequencing platform and contained 11 cerebral cortex tissues from 8‐day‐old (P8) mice with HIBD and sham mice separately at three various time points (3, 8, and 24 h). And the second data set GSE144456 retrieved from the GPL10333 sequencing platform, included 24 cerebral cortex samples from HIBD mice and eight samples from sham mice at four time points (3, 6, 12, and 24 h). In this data set, 5‐day‐old (P5) mice and 10‐day‐old (P10) mice imitate the state of premature infant HIBD and term infant HIBD respectively. Table 1 lists the detailed information on the GSE23317 and GSE144456 data sets.

**Table 1:** The detail information of data sets.

Data set	Organism	Tissue	Platform	Samples (Control/HIBD)	Type
GSE23317	*Mus musculus*	Cerebral cortex	GPL6885	P8 3 h:4/3 8 h:3/4 24 h:4/4	mRNA
GSE144456	*Mus musculus*	Forebrain	GPL10333	P5, P10 3 h: 3/3 6 h: 3/3 12 h: 3/3 24 h: 3/3	mRNA

Abbreviations: HIBD, hypoxic‐ischemic brain damage; mRNA, messenger RNA.

DEGs were screened using the “limma” R package^15^ (version 3.58.1). The filtering criteria were |log2FC | > 0.3 and *p* < .05. Then, OS‐related genes with a relevance score > 6.5 were acquired from the Genecards database^16^ using the keyword “oxidative stress.” OS‐DEGs were identified by intersecting OS‐related genes with DEGs in GSE23317. Additionally, the heatmap of the OS‐DEGs was drawn utilizing the “pheatmap” R package (version 1.0.12).

### 2.2 Gene Ontology (GO) and Kyoto Encyclopedia of Genes and Genomes (KEGG) enrichment analysis

GO and KEGG enrichment analysis were conducted to determine the molecular pathways and function of OS‐DEGs in neonatal mouse HIBD using the “ClusterProfiler” R package^17^ (version 4.10.1). GO analysis is a well‐known method forannotating the cellular components (CC), molecular functions (MF), and biological processes (BP) of genes. KEGG analysis is a common approach to predict the potential signaling pathways in which genes may be involved.

### 2.3 Gene Set Enrichment Analysis (GSEA)

GSEA^18^ was conducted to explore the overall level of gene expression profiles in GSE23317 data set using the “clusterProfiler” R package (version 4.10.1), and GSEA was performed with all genes. Single‐gene GSEA was used to investigate the pathways related to the four optimal genes.

### 2.4 Protein−protein interaction (PPI) network analysis and hub genes validation

The STRING (http://string-db.org/) database is a well‐known online database which is used to construct PPI networks. The STRING database was utilized to construct the PPI network of OS‐DEGs. And then, the network was visualized using Cytoscape software^19^ (version 3.9.1). The top 10 hub genes were identified using the CytoHubba^20^ plugin and degree algorithm of the Cytoscape software. Ultimately, the top 10 hub genes were further verified in the GSE144456 data set, and then the identified genes were defined as the optimal OS‐DEGs.

### 2.5 Evaluation of immune cell infiltration

CIBERSORT^21^ (version 0.1.0) was used to investigate the infiltration of immunocytes into HIBD and control samples using a mouse feature matrix,^22^ a mouse‐specific leukocyte signature matrix containing 25 immunocytes and 511 immunomarker genes. A boxplot was plotted of the differentially expressed immune cells between the two groups. Furthermore, the correlation between immunocyte subgroups and gene expression levels were analyzed using Spearman's rank correlation in this study, and a heatmap of the correlation analysis between immune cells and the optimal OS‐DEGs was drawn.

### 2.6 Drug‐gene interaction

DGIdb is a drug‐gene interaction database which is used to predict potential gene‐targeted drugs. A network of genes and predicted drugs was visualized using Cytoscape software.

#### 2.6.1 Construction of HIBD rat model

The Rice‐Vannucci method^23^ was used to establish a neonatal rat HIBD model. Six 7‐day‐old SD rats weighing 10−20 g were randomly allocated to each groups using the random number table. Under‐ or overweight rats were excluded from the experiment. The rats were healthy, with no genetic modifications. In control samples, the unilateral common carotid artery of neonatal rats was separated without ligation. For the HIBD samples, the same artery of the neonatal rats was isolated and cut from the middle after ligation with a double femoral 4−0 suture. The operative time did not exceed 5 min. After surgery, the newborn rats were placed back in their mother's cage for 60 min. The neonatal rats were then placed in a home‐made hypoxic box. The nitrogen and oxygen mixed gas (8%) 0_2_ + 92% N_2_ was used as the input. An oxygen detection probe was used to maintain the oxygen concentration at about 8%, and the temperature was controlled at 36 + 1°C for 2.5 h. After exposure to hypoxia, the newborn rats were placed back to the nest. After 24 h of modeling, the rat cerebral cortex tissue was obtained under anesthesia. Animal experiment was approved by the Laboratory Animal Welfare Ethics Administrative Committee of Sun Yat‐sen University (Number: 2023001659).

### 2.7 Quantitative reverse transcription polymerase chain reaction (RT‐qPCR)

Left cerebral cortical tissue was obtained from neonatal rats after 24 h of hypoxia and ischemia. The brain tissue was weighed and added to Lysis Buffer, and fully homogenized with a grinder. The RNA extraction kit, cDNA synthesis kit and SYBR were purchased from EZScience Biotechnology Corporation (EZBioscience). The primer sequences for the target gene list in Supporting Information S1: Table 1. β‐actin was acted as an internal reference. And all experimental data were calculated using the $2^{-\Delta \Delta C_{t}}$ method.^24^

### 2.8 Immunohistochemistry

Immunohistochemistry experiments were conducted to detect the expression of the optimal genes (Jun，Fos, Tlr2, and Atf3) in rat HIBD samples and sham samples. Firstly, the slices were treated with xylene and rehydrated with ethanol. The slices were then placed in a hydrogen peroxide solution, boiled in a sodium citrate buffer solution, and blocked in 5% BSA at indoor temperature. Then, the sections were incubated with a rabbit anti‐rat JUN antibody (1:300 dilution, ab40766, Abcam), rabbit anti‐rat FOS antibody (1:300 dilution, AF5354, Affinity Biosciences), rabbit anti‐rat TLR2 antibody (1:200 dilution, 17236‐1‐AP, Proteintec) and rabbit anti‐rat ATF3 polyclonal antibody (1:300 dilution, ab305293, Abcam) 4°C overnight. On the second day, the secondary antibodies were incubated at indoor temperature, and DAB solution was added. Hematoxylin was used for counterstaining and the sections were washed with hydrochloric acid and with water. Finally, the sections were sealed after gradient dehydration and transparency, and observed under a light microscope, and imaged.

#### 2.8.1 Statistical analysis

Data processing was performed using R software (version 4.2.3). Student's *t*‐test was conducted to compare the differences between the two groups using Graphpad Prism 9. The results of RT‐qPCR were exhibited with mean and SD. *p* < .05 was regarded as significantly changed.

## 3 RESULT

### 3.1 Screening of OS‐DEGs

**Figure 1:**
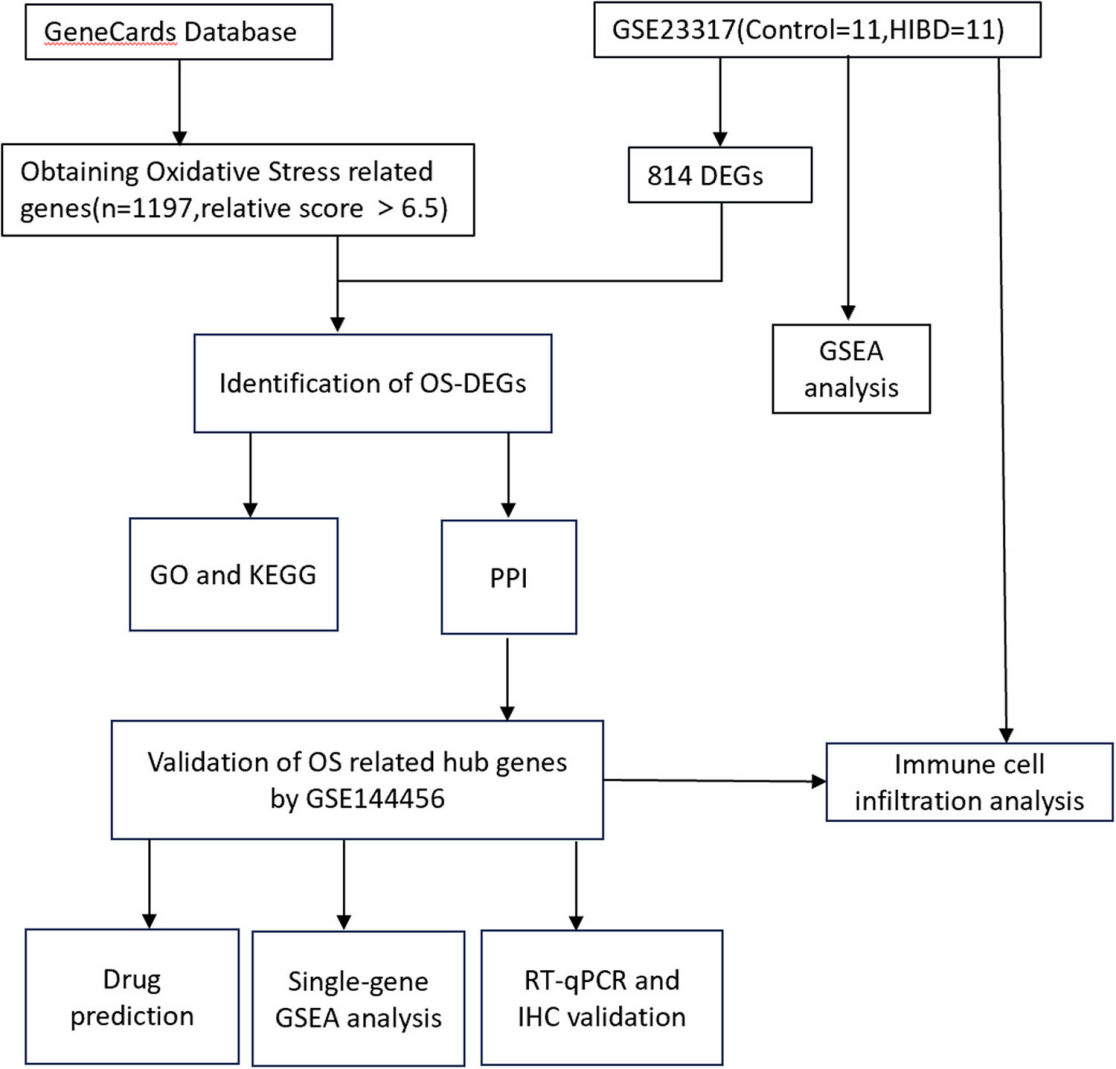
The workflow of this research.

**Figure 2:**
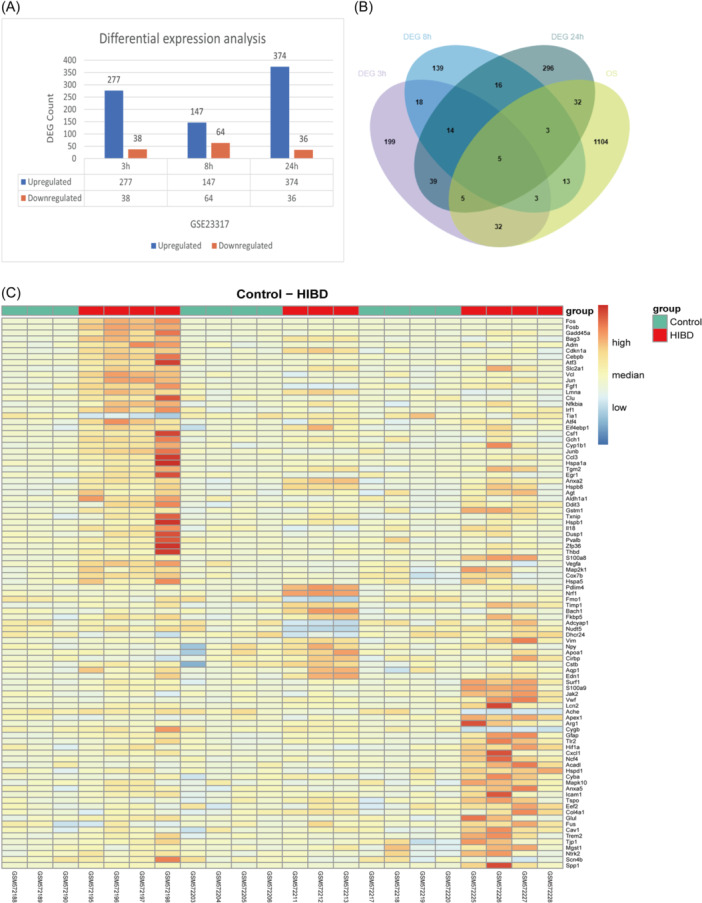
Differential gene screening. (A): Differentially expressed of genes after 3, 8, and 24 h of HIBD modeling. (B): Venn diagram showing the relationship of OS‐related genes and DEGs at different time points. (C): Heatmap of the 93 OS‐DEGs. DEGs, Differentially expressed genes screened from GSE23317; HIBD, hypoxic‐ischemic brain damage; OS‐DEGs, oxidative stress‐related DEGs.

The workflow is illustrated in Figure 1. As is illustrated in the differential expression plot (Figure 2A), 315, 211, and 410 DEGs were obtained in the data set GSE23317, based on the comparison of gene expression between the control and HIBD samples at 3, 8, and 24 h, respectively. After removing overlapping genes, 814 DEGs were identified as DEGs, including 676 upregulated genes and 138 downregulated genes. A total of 1197 OS‐related genes with a correlation score > 6.5 were selected from the GeneCards database. These were further intersected with DEGs and a number of 93 OS‐DEGs were identified. A Venn diagram and heatmap of the 93 OS‐DEGs are shown in Figure 2B,C, respectively.

### 3.2 Enrichment analysis of OS‐DEGs

**Figure 3:**
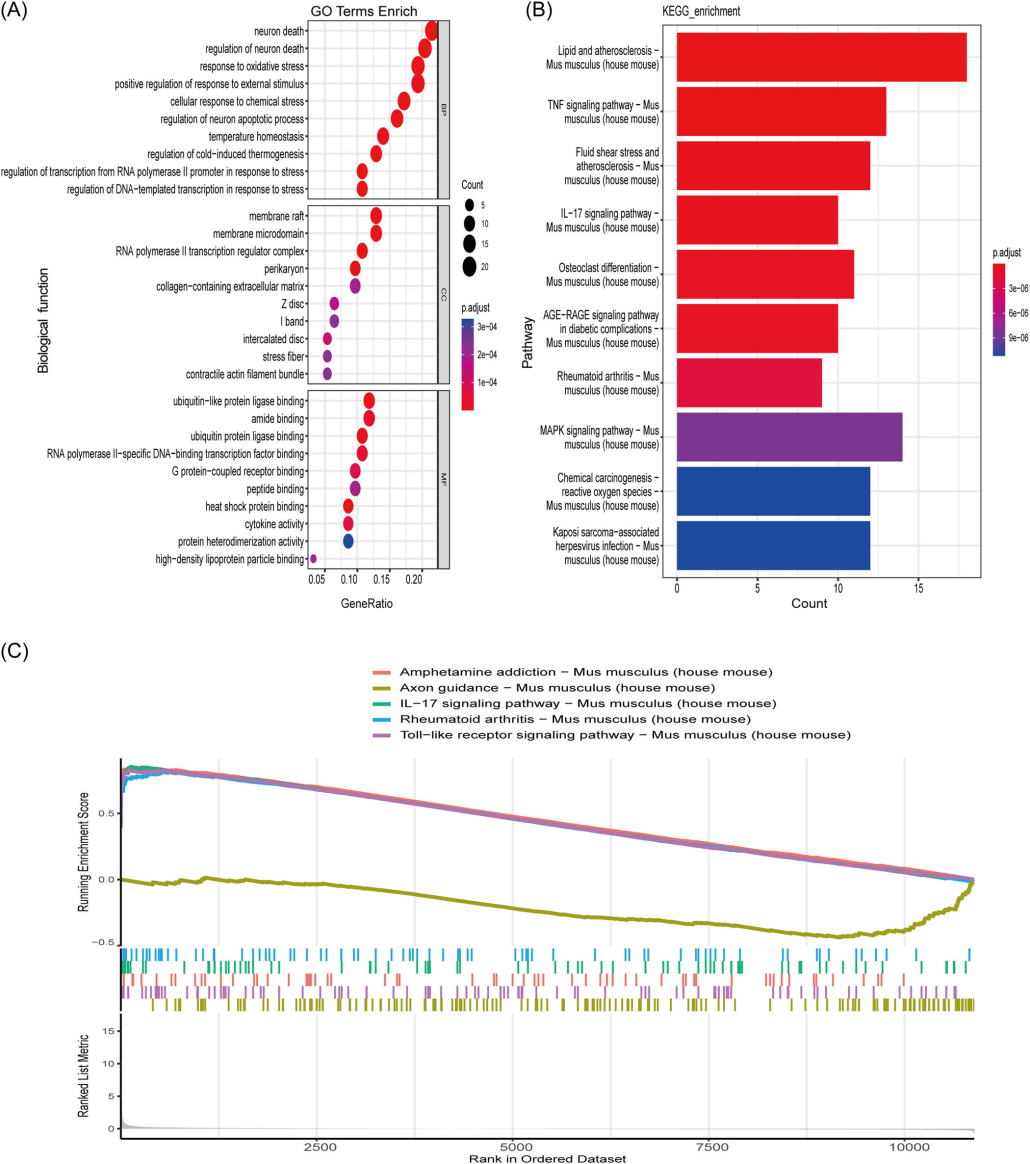
Evaluation of functional enrichment. (A): GO analysis of OS‐DEGs, including BP, CC, MF. (B): KEGG analysis showed top 10 pathways of the OS‐DEGs. (C): GSEA analysis showed top five signaling pathways in GSE23317 data set. BP, biological processes; CC, cellular components; DEGs, Differentially expressed genes screened from GSE23317; GO, Gene Ontology; HIBD, hypoxic‐ischemic brain damage; MF, molecular functions; OS‐DEGs, oxidative stress‐related DEGs.

The 93 OS‐DEGs were subjected to enrichment annotation analysis. GO analysis (Figure 3A) indicated that for BP, OS‐DEGs were primarily enriched in neuronal death, OS, positive regulation of response to external stimuli, and cellular response to chemical stress, etc. For CC, OS‐DEGs were considerably abundant in the perikaryon, RNA polymerase Ⅱ transcription regulator complex, membrane microdomains, and membrane rafts, etc. In MF, OS‐DEGs were primarily enriched in G protein‐coupled receptor binding, RNA polymerase Ⅱ‐specific DNA‐binding transcription factor binding, ubiquitin protein ligase binding, amide binding, and ubiquitin‐like protein ligase binding. KEGG enrichment analysis suggested that OS‐DEGs tended to be highly involved in the lipid and atherosclerosis, TNF, ROS, and IL‐17 pathways (Figure 3B). GSEA analysis was performed to further investigate the underlying biological pathways and processes of all genes in HIBD and sham samples. The GSE23317 data set suggested that the top five pathways involving the most genes were the IL‐17 signaling pathway, Toll‐like receptor signaling pathway, axon guidance, amphetamine addiction, and rheumatold arthritis pathways (Figure 3C).

### 3.3 PPI network construction and hub genes validation

**Figure 4:**
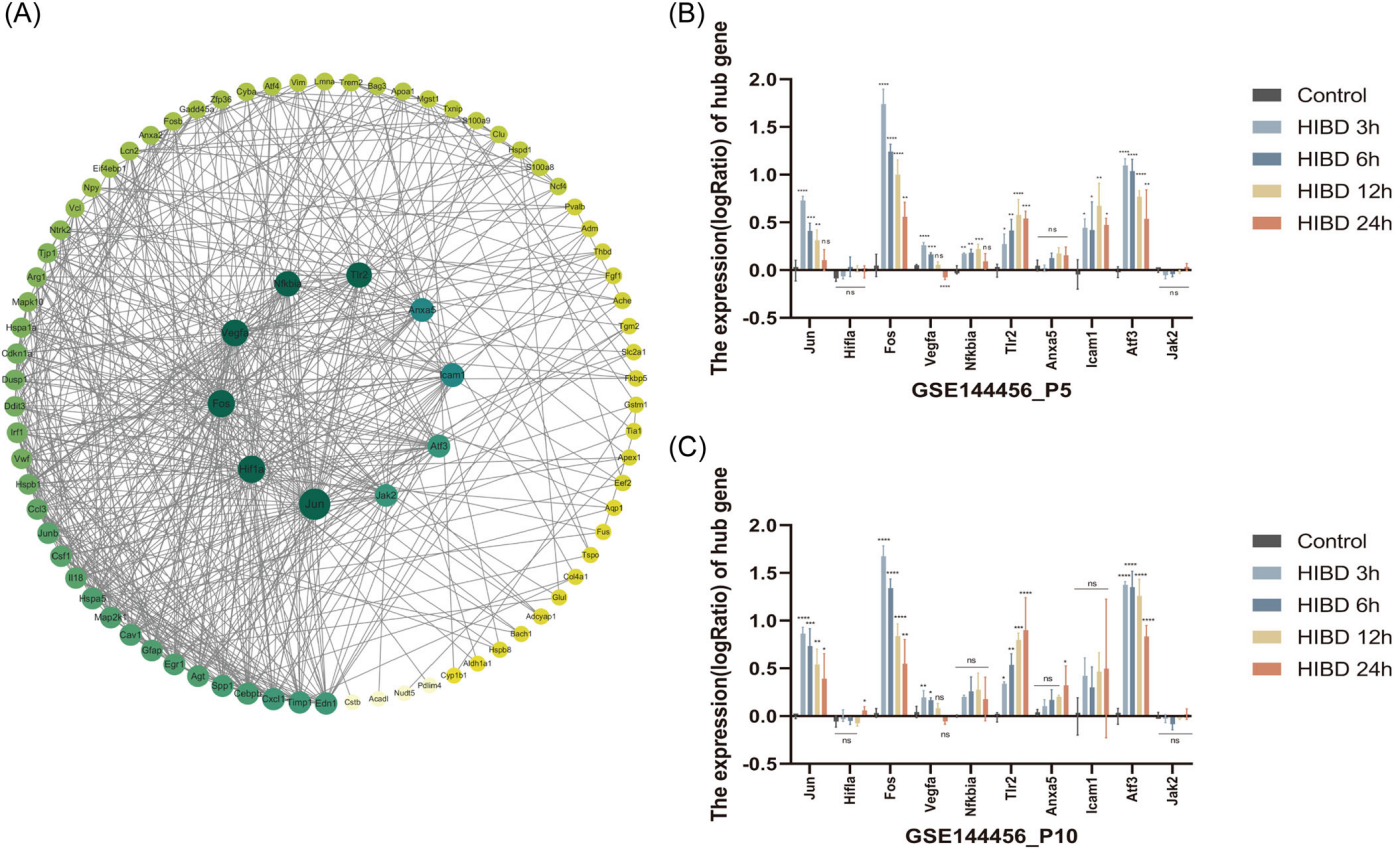
Screening of oxidative stress hub genes (A): PPI network of 84 OS‐DEG. (B, C): Expression levels of hub OS‐DEGs in mouse models of HIBD at different time points. PPI = protein‐protein interactions. ****(*p* ≤ .0001), ***(*p* ≤ .001), **(*p* ≤ .01) and *(*p* ≤ .05). DEGs, Differentially expressed genes screened from GSE23317; HIBD, hypoxic‐ischemic brain damage; OS‐DEGs, oxidative stress‐related DEGs; PPI, Protein−protein interaction.

A total of 93 OS‐DEGs were used to construct the PPI network using STRING database and the Cytoscape program. Excluding the disconnected nodes in the network, the PPI network included 84 nodes and 533 edges (Figure 4A). Using the degree algorithm, the top 10 hub genes (Jun, Hifla, Fos, Vegfa, Nfkbia, Tlr2, Anaxa5, Icam1, Atf3, and Jak2) were identified. The GSE144456 data set was used to validate the expression of the top 10 hub OS‐DEGs. The histograms (Figure 4B,C) showed that the expression of these hub genes in mouse cerebral cortex cells at different ages, indicating that the four hub OS‐DEGs (Jun, Fos, Tlr2, and Atf3) were differentially expressed at P5 and P10. The expression trends were consistent with those of the GSE23317, indicating that the results were comparatively reliable. Thus, the four OS‐DEGs (Jun, Fos, Tlr2, and Atf3) were defined as the optimal OS‐DEGs and used for further analysis.

### 3.4 Single‐gene GSEA analysis

**Figure 5:**
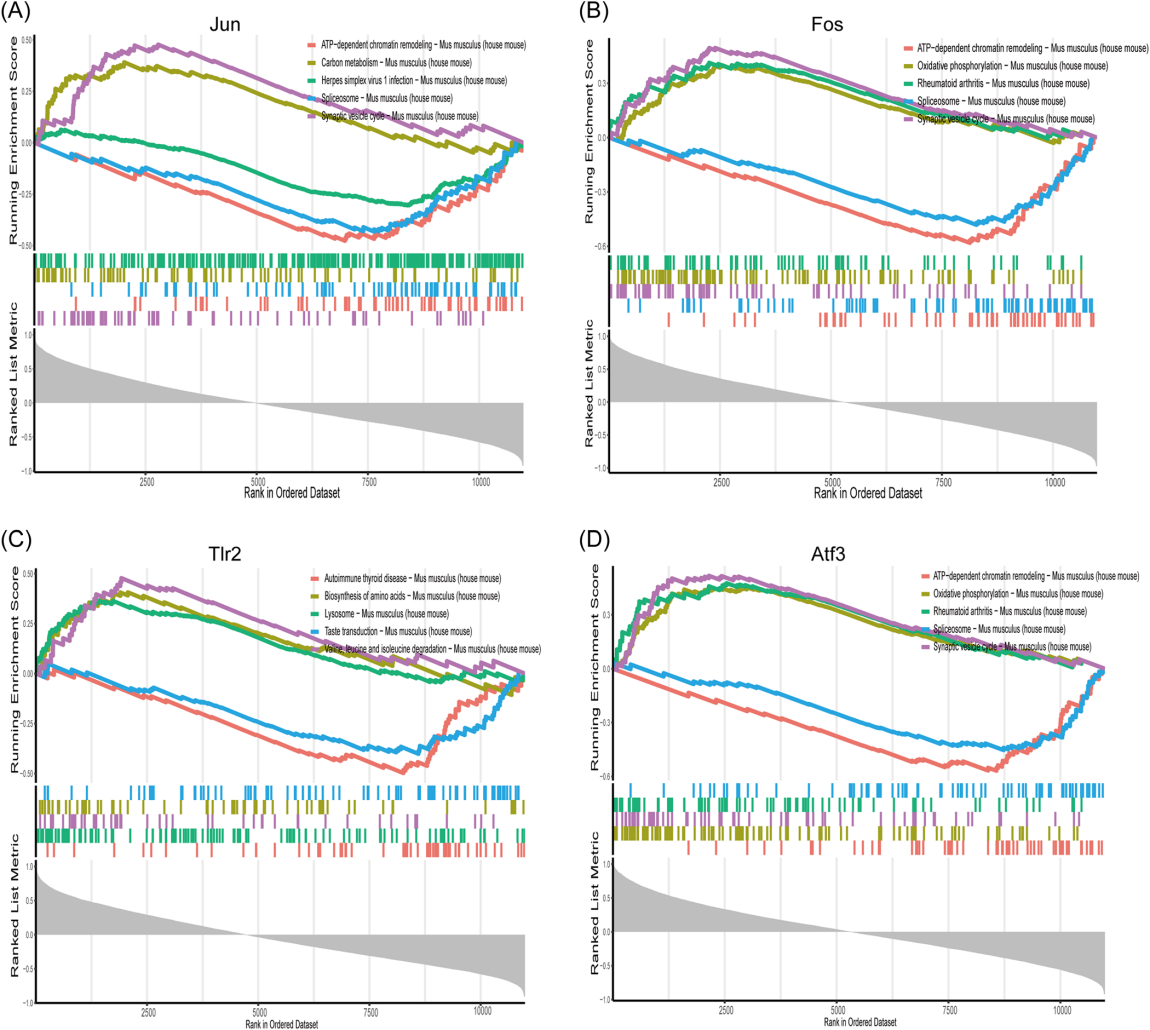
Single‐gene KEGG‐GSEA analysis in Jun (A), Fos (B), Tlr2 (C), and Atf3 (D). GSEA, Gene Set Enrichment Analysis; KEGG, Kyoto Encyclopedia of Genes and Genomes.

Single‐gene GSEA‐KEGG analysis was further conducted to investigate the underlying signaling pathways related to each optimal gene in HIBD. The top five signaling pathways for each marker gene are shown in Figure 5. The results showed that these marker genes were mainly involved in adenosine triphosphate ‐dependent chromatin remodeling; oxidative phosphorylation; carbon metabolism; synaptic vesicle cycle; spliceosomes; biosynthesis of amino acids; valine; leucine and isoleucine degradation; and lysosomes.

### 3.5 Mice immune infiltration analysis

**Figure 6:**
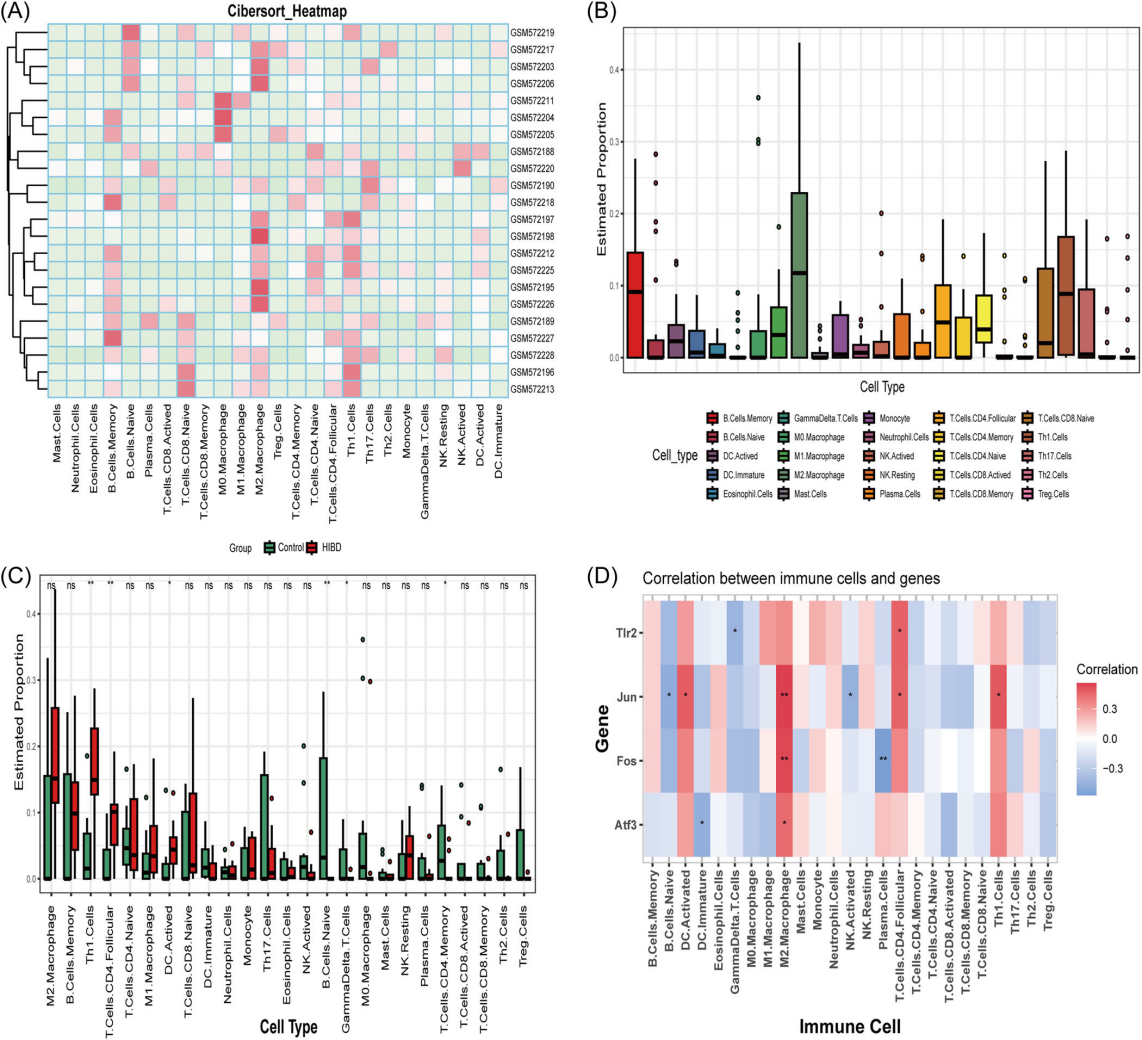
Immune cell and OS‐DEGs relevance analysis. (A) The heat diagram of 25 immunocyte subgroups in different samples; (B) Infiltrated proportion of 25 immunocyte subgroups; (C) The expression differences of immune cell in control samples and HIBD samples; (D) Relationship between the optimal OS‐DEGs and immunocytes. **(*p* ≤ .01), *(*p* ≤ .05) and ns (*p* > .05). HIBD, hypoxic‐ischemic brain damage; OS‐DEGs, oxidative stress‐related DEGs.

Using CIBERSORT with a mouse feature matrix, the expression and infiltration abundances of the 25 different mouse immune cells in different samples are shown in a heatmap (Figure 6A) and a boxplot (Figure 6B). Moreover, the diagram (Figure 6C) shows the expression of immune cells in different groups, and reveals that six immunocyte subgroups in HIBD samples were strongly distinct from the control samples. The proportion of T‐helper (Th)1 cells, activated dendritic cells (DC), and follicular CD4^+^ T cells were upregulated in the HIBD group; however, the proportions of memory CD4^+^ T Cells, gamma delta T Cells, and naive B Cells were downregulated (*p* < .05). Spearman's correlation analysis (Figure 6D) of immune cells and genes suggested that Jun, Fos, and Atf3 all had a higher positive correlation with M2 macrophages. Tlr2 expression negatively correlated with Gamma‐Delta T cells, and positively correlated with follicular CD4^+^ T cells. Activated DC, follicular CD4^+^ T cells, and Th 1 Cells all had a positive correlation with Jun. Jun expression had a negatively correlated with Naïve B cells and activated NK cells. Fos was negatively correlated with plasma cells, and Atf3 was negatively correlatedd with immature DC cells. These results suggested that the optimal OS‐DEGs may influence the immune cell infiltration and the immune microenvironment in mice with HIBD.

### 3.6 Drug prediction

**Figure 7:**
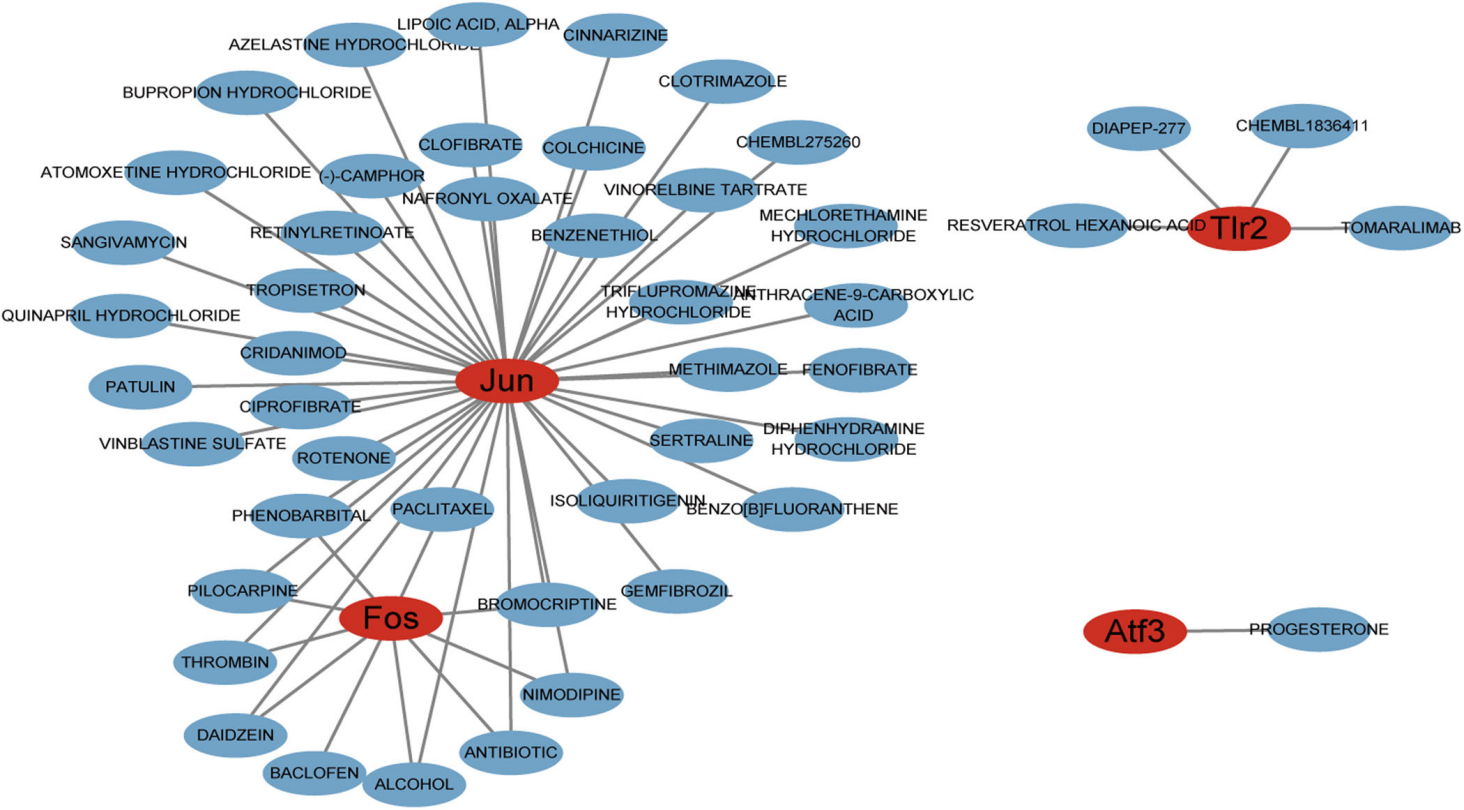
Prediction of gene‐targeted drugs.

Using the DGIdb database, we identified underlying drugs that might affect the marker genes. Interactions between the four genes and drugs were visualized using Cytoscape program (Figure 7). A total of 47 drugs were screened, including 42 for Jun, 10 for Fos, four for Tlr2, and one for Atf3. Among these, Tomaralimab was the first humanized Tlr2 inhibitor. Atf3 is a progesterone regulated gene. Significantly, some of the predicted drugs, such as alpha‐lipoic acid, antibiotics (anisomycin^25^), and thrombin, play a vital role in alleviating brain damage in HIBD.

### 3.7 Confirmation of the expression level of four optimal hub genes

**Figure 8:**
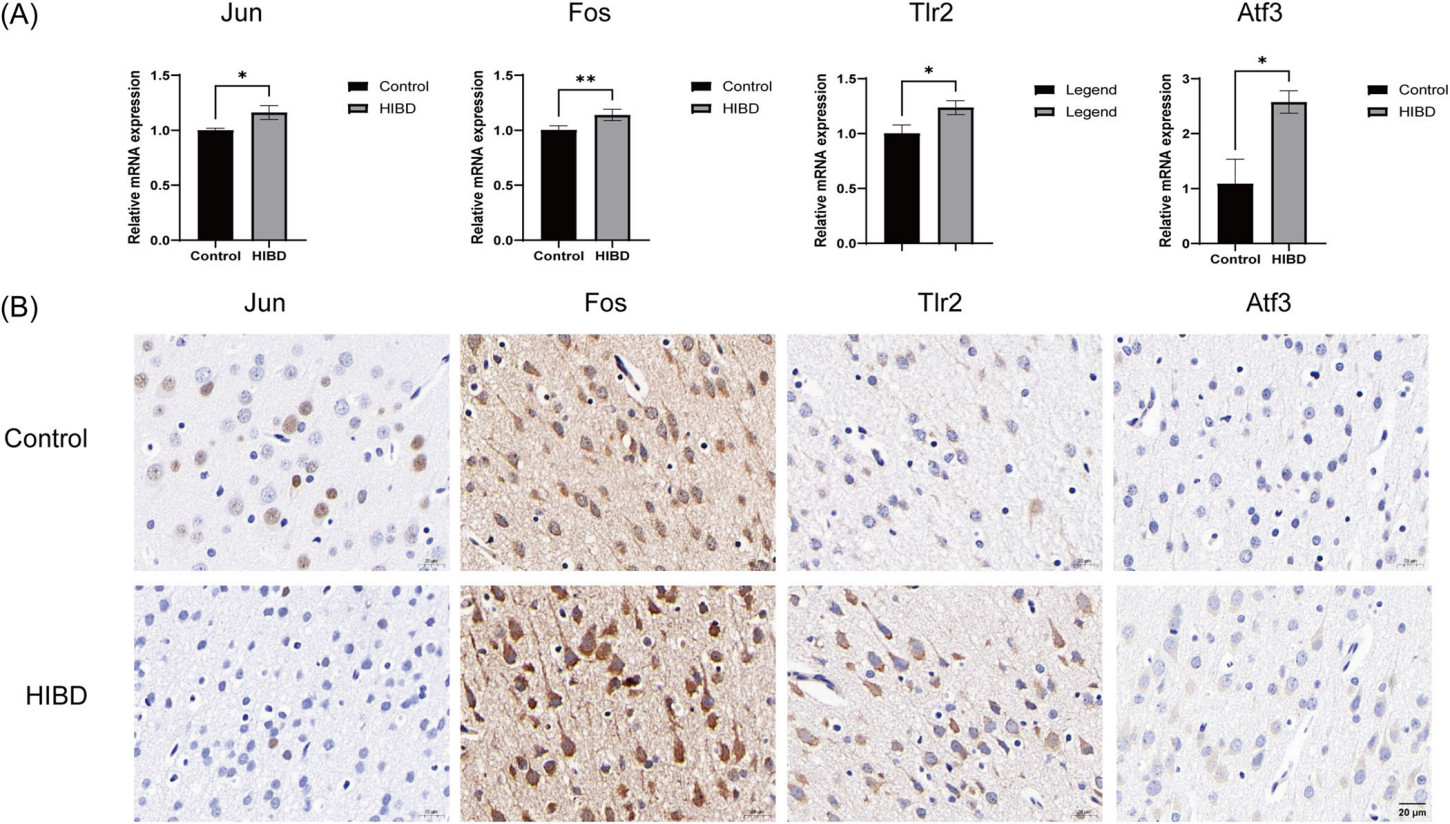
Confirmation of the expression of optimal OS‐DEGs in rat HIBD model. (A): The RT‐qPCR results of Jun, Fos, Tlr2 and Atf3 in rat tissues (*n* = 3). (B): Immunohistochemistry validation of Jun, Fos, Tlr2 and Atf3 in rat samples (*n* = 3). **(*p* ≤ .01), *(*p* ≤ .05). The scales are 20 μm. HIBD, hypoxic‐ischemic brain damage; OS‐DEGs, oxidative stress‐related DEGs; RT‐qPCR, quantitative reverse transcription polymerase chain reaction.

The mRNA expression levels of the four optimal OS‐DEGs were validated in neonatal rat HIBD samples and control samples using RT‐qPCR. Jun, Fos, Tlr2, and Atf3 were upregulated in HIBD samples compared with the control samples (Figure 8A). Then, immunohistochemistry experiment was conducted to further detect the expression levels of the four OS‐DEGs. The results (Figure 8B) showed that Fos, Tlr2 and Atf3 were upregulated in HIBD tissues compared with control tissues, whereas Jun was downregulated in HIBD compared to the control tissue.

## 4 DISCUSSION

The mechanism underlying HIBD are complex. OS and inflammation interact in a complicated manner, resulting in unremitting brain damage in the neonatal patients with HIBD. Recent research have suggested that OS‐targeting drugs, such as dihydroartemisinin and irisin, can ameliorate the brain damage in neonatal rat HIBD by decreasing OS and inhibiting inflammation. Moreover, persistent detection of the central nervous system (CNS) in neonatal HIBD suggests a substantial reduction in reduced GSH, an important intracellular antioxidant and a significant determinant of CNS damage after the occurrence of hypoxic‐ischemic event.^26^ Collectively, these results suggest that reducing OS in HIBD is a promising treatment strategy. However, few studies have investigated the OS‐related genes in HIBD progression. Thus, this study aimed to explore the OS related genes in neonatal HIBD and investigate the correlation between these genes and immunocytes.

Based on the analysis of differential gene expression in HIBD and OS‐related genes, 93 OS‐DEGs were screened. Different enrichment methods were used to further investigate the impact of OS‐DEGs on HIBD. GO annotation analysis showed that the OS‐DEGs were mainly enriched in neuronal death, OS, positive regulation of responses to external stimuli, cellular responses to chemical stress, and apoptosis. KEGG enrichment analysis suggested that OS‐DEGs tended to be highly involved in several inflammatory signaling pathways, including the TNF, ROS, and IL‐17 signaling pathways. GSEA analysis further revealed that the top five pathways involving most genes were the IL‐17 signaling pathway, Toll‐like receptor signaling, axon guidance, amphetamine addiction, and rheumatold arthritis pathways. Consistent with the results of our work, previous studies have revealed some signaling pathways involved in the pathogenesis of HIBD, ROS,^27^ TNF,^28^ Toll‐like receptor signaling, and IL‐17 signaling pathways. During HIBD, ROS levels cannot be rapidly decreased by the antioxidant systems because of the disorganized oxidative metabolism, leading to an excessive ROS accumulation.^29^ The excessive ROS can cause the release of proinflammatory cytokine and activate microglia, and conversely, microglia can secrete free radicals and proinflammatory cytokines (TNF‐α, IL‐1β, etc.).^30^ High concentrations of these cytokines under OS are positively correlated with HIBD severity.^31^ IL‐17 is a cytokine produced by T helper 17 cells. In a nonhuman primate model of HIBD, the serum level of IL‐17 has decreased in the moderate/severe injury groups compared with the normal/mild groups.^32^ Toll‐like receptors (TLRs) are innate immune proteins that play a significant role in cerebral ischemia. Studies have confirmed that inhibition of TLR4 can improve the proinflammatory situation, attenuate OS and finally ameliorate brain damage in HIBD.^33^ A clinical study has showed that TLR‐3 expression alters in response to white matter damage in preterm neonates, suggesting that TLR‐3 may be involved in the progress of brain.^34^ Furthermore, studies have shown that the densities of immature granule cells during axon guidance and outgrowth were increased in the brain sections of neonatal HIE.^35^

PPI network analysis identified 10 hub genes: Jun, Hifla, Fos, Vegfa, Nfkbia, Tlr2, Anaxa5, Icam1, Atf3, and Jak2, which were validated using the GSE144456 data set. Four hub genes (Jun, Fos, Tlr2, and Atf3) showed consistent expression trends in preterm and term mice with HIBD. The oncogenes c‐Jun is a transcription factor belonging to the activator protein‐1 family. Studies have shown that c‐Jun is associated with cell death either in response to cellular injury or under antiproliferative conditions.^36^ Fos is a transcription factor that can modulate the inflammatory response by inhibiting nuclear factor kappa B (NF‐κB) activity.^37^ In addition, studies have shown that Fos, a gene reflecting neuronal activity, is activated in neurons and involved in neuroprotective and anti‐inflammatory effects after HIBD.^38^, ^39^ Tlr2 belongs to the Toll‐like receptor family, which senses and responds to different stimuli from microbial pathogens and damaged cells. Tlr2 knockout can reverse the decrease in total antioxidant capacity in lipopolysaccharide‐induced granule cells and the increase in malondialdehyde, attenuating cellular OS.^40^ Atf3 is a stress‐regulating transcription factor that controls metabolism, immunity, and a variety of extracellular signals.^41^ Silencing Atf3 expression reduces OS in OGD‐treated HL‐1 cells.^42^ In addition, the upregulation of Atf3 in damaged neurons can decrease the deaths of hippocampal neuronal death.^43^ Overall, the four hub OS‐genes participated in HIBD progression.

Many studies have shown the importance of inflammatory reactions in the pathogenesis of neonatal HIBD, and the dysregulation of immunocytes is relevant to the severity of outcome in neonatal patients.^44^ Our study revealed the dysregulation of six immunocyte subgroups, comprising activated DC cells, follicular CD4^+^ T cells, Th1 cells, memory CD4^+^ T cells, Gamma‐Delta T cells and naive B cells in neonatal mouse HIBD samples. In addition, the correlation analysis of genes and immunocytes has revealed that the expression of Jun, Fos and Atf3 positively correlated with M2 macrophages. Tlr2 had a negative correlation with γδ T cells, and a positive correlation with follicular CD4^+^ T cells. Jun expression also positively correlated with activated DC, follicular CD4^+^ T cells, and Th 1 cells. Various immune cells are closely associated with ischemic damage in HIBD, including T cells, DC, B cells and macrophages.^45^ Research has shown that hypoxic‐ischemic induced brain damage can trigger obvious permeation of CD4^+^ T cells in the impaired sites and an increase in Th1 cells‐mediated proinflammatory shift, and the proinflammatory situation is closely associated with poor short‐term behavioral consequences.^46^ In addition, CD4^+^ helper T cells can induce the release of IFN‐γ which directly result in neuronal death, and γδ T cells are the primary origination of IL‐17 after the occurrence of a hypoxic‐ischemic event.^47^ The depletion of γδT cells can provide protection in the hypoxic‐ischemic mouse model.^48^ DC are an antigen‐presenting cell recognized by T‐cells and related to the translocation of NF‐κB (an inflammatory transcription factor).^49^ B cells are reportedly involved in dementia and cognitive impairment after adult stroke; however, their role in B cells in neonatal HIBD has not been widely explored.^50^ These findings further indicate that immunity is tightly associated with the development of HIBD.

Hypothermia is the only approved therapy method for neonatal HIBD patients. However, some neonatal patients remain at risk of irreversible neurological sequelae or death. Therefore, additional therapeutic strategies are urgently needed. A total number of 47 drugs that targeting four OS‐related genes were identified, and these drugs may reduce the sequelae when used in conjunction with hypothermia. Previous studies have shown that some drugs, such as alpha‐lipoic acid^51^ and thrombin,^52^ play essential roles in antagonizing brain damage in HIBD. However, further investigations are required to explore the exact mechanisms of genes and gene‐targeted drugs.

RT‐qPCR and immunohistochemistry validation tests were preformed to detect the expression levels of the four marker genes in rat samples. RT‐qPCR results suggested that Jun, Fos, Tlr2 and Atf3 were significantly upregulated in HIBD samples. Immunohistochemistry results showed that Fos, Tlr2, and Atf3 were upregulated in HIBD rat tissues compared with the control tissues, whereas Jun was downregulated. Further research is required to explore the underlying mechanisms.

The study had a few limitations. First, the two data sets obtained from GEO were at slightly different time points, and the ages of the mouse pups in the two data sets were slightly different. Second, the bioinformatics analysis was performed using mouse data sets, whereas validation in this study was performed using rat pups. Third, the definite mechanisms underlying OS‐related genes and immune cell infiltration require further exploration. Although, bioinformatics analysis of microarrays can be useful for screening the potential therapeutic targets of HIBD, further research are necessary to identify the biological significance and detailed molecular mechanisms of OS‐related genes in HIBD based on clinical prognosis and the time of onset.

## 5 CONCLUSION

To our knowledge, this is the first to study the underlying OS‐related genes in HIBD and their correlation with immunocytes infiltration. Three potential OS‐related marker genes (Fos, Tlr2, and Atf3) associated with HIBD were identified using bioinformatics analysis and experimentally validated in rat samples. These OS‐related genes may serve as underlying biomarkers and therapeutic targets for OS in HIBD. This study is conducive to help us understanding the mechanisms of OS and providing novel insights into the progress and treatment of HIBD.

## AUTHOR CONTRIBUTIONS

**Ni Jin**: Project designation, writing original draft, data analysis, conducting RT‐qPCR and IHC experiments. **Sha Sha**: Writing the revised draft and constructing the rat HIBD model. **Yanghao Ruan**: Constructing the rat HIBD model. **Ying Ouyang**: Providing writing guidance.

## Supporting information

Supporting information.

## Data Availability

The data that support the findings of this study are available from the corresponding author.
