# Supplementary figures and images for: Identification and analysis of oxidative stress‐related genes in hypoxic‐ischemic brain damage using bioinformatics and experimental verification
Source: Immun Inflamm Dis. 2024 Aug 22;12(8):e70000. doi: 10.1002/iid3.70000 (PMC11340634; doi:10.1002/iid3.70000)

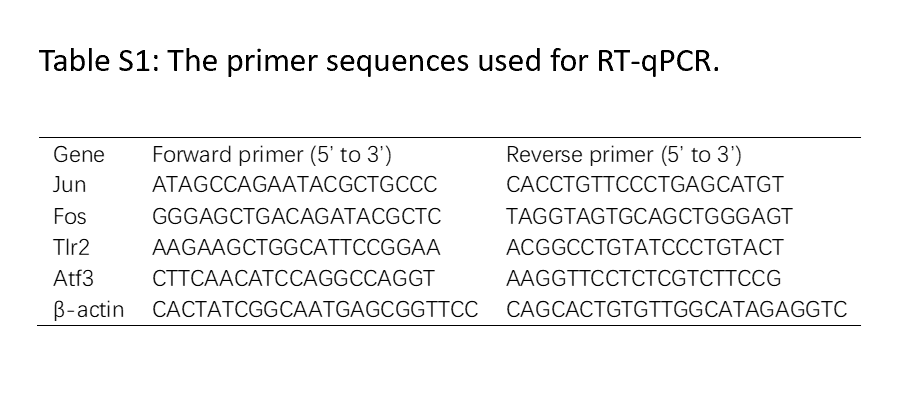

Supplement: Supplementary file 1 — Supporting information. [file IID3-12-e70000-s001.docx]
